# Integration of QTL mapping and GWAS reveals the complicated genetic architecture of chemical composition traits in tobacco leaves

**DOI:** 10.3389/fpls.2025.1616591

**Published:** 2025-06-25

**Authors:** Asad Ullah, Zhijun Tong, Muhammad Kamran, Feng Lin, Tianneng Zhu, Muhammad Shahzad, Umaira, Xuejun Chen, Bingguang Xiao, Haiming Xu

**Affiliations:** ^1^ Institute of Crop Science, College of Agriculture and Biotechnology, Zhejiang University, Zhejiang, Hangzhou, China; ^2^ Institute of Bioinformatics, College of Agriculture and Biotechnology, Zhejiang University, Zhejiang, Hangzhou, China; ^3^ Key Laboratory of Tobacco Biotechnological Breeding, National Tobacco Genetic Engineering Research Center, Yunnan Academy of Tobacco Agricultural Sciences, Yunnan, Kunming, China

**Keywords:** tobacco leaf chemistry, QTL mapping, QTL by environment interaction, epistasis, genome wide association study, GO and KEGG enrichment, candidate gene

## Abstract

Tobacco (*Nicotiana tabacum L.*) is a significant industrial crop whose leaves serve as the primary raw material for various smoking products. However, the genetic basis of tobacco leaf chemical composition which is a key factor in product quality, remains largely unexplored. To address this, a QTL study was undertaken to pinpoint genomic loci associated with 21 leaf chemistry traits using a recombinant inbred line population of 271 genotypes evaluated across multiple environments. Variance components and heritability were estimated for nine multi-environment phenotypes. Phenotypic correlations between paired traits were calculated within each environment, while genotypic correlations were estimated across multi-environment phenotypes. Mixed-linear-model-based composite interval mapping (MCIM) was employed using *QTLNetwork*, leading to the identification of 18 QTLs with significant individual effects. Among these, *qPA15-18* and *qGA15-18* exhibited pleiotropic effects, while three epistatic QTL pairs associated with chlorogenic acid (CHA) and rutin (RU) were also detected. Notably, no significant QTL-by-environment interactions were observed. Through integration of association mapping, bioinformatics analysis and gene enrichment analysis of the QTL regions, we predicted three candidate genes. *Nt08g00266* and *Nt22g03479* were identified as pleiotropic genes associated with starch and total sugar, and with total sugar and reducing sugar, respectively. While, *Nt16g00236* exhibited significant association with total plant alkaloid. This study lays the groundwork for tobacco varieties with enhanced chemical composition by targeting the identified QTLs and candidate genes, ultimately contributing to production of higher-quality smoking products.

## Graphical Abstract

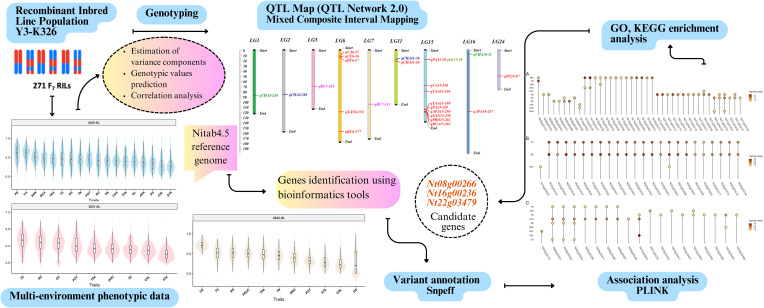

## 1 Introduction

Tobacco (*Nicotiana Tabaccum L.*) is an allopolyploid species (2n = 48) that originated from interspecific hybridization between *N. Sylvestris* (2n=24) and *N. tomentosiformis* (2n=24) (Tong et al., 2020, 2021). It is predominantly known for its leaves, which are used in the production of various smoking products intended to be chewed, snuffed, sucked, or smoked (Li et al., 2023). The quality of these smoking products and tobacco yield largely depends on the chemical composition of their leaves (Li et al., 2023). Tobacco is a chemically complex plant in which approximately 3000 chemical constituents have been identified and characterized in its leaves and around 4000 in its smoke (Leffingwell, 1999). These chemical constituents provide the framework for tobacco leaf chemistry and differentiate different tobacco types like flue-cured, air-cured, and oriental (Leffingwell, 1999; Ji et al., 2024).

Among the key chemical traits in tobacco, total plant alkaloids primarily nicotine, range from 0.5% to 8%, within the main cultivars*, N. tabacum* and *N. rustica*. Alkaloids define stimulating properties and positively correlate with taste and smoking density (Leffingwell, 1999). Besides the alkaloids, carbohydrates are the main component in determining the smoke quality (Stedman, 1968). Sugars (reducing sugar, total sugar) constitute 10–20% of dry leaf matter and enhance the flavor by adding sweetness and mitigating the harshness of nicotine and other alkaloids (Talhout et al., 2006; Tong et al., 2025). Cellulose is more concentrated in the midrib than in the lamina of the leaf and provides structural stability (Stedman, 1968). However, excessive cellulose imparts an unpleasant, burnt paper-like taste (Leffingwell, 1999). Pectin not only strengthens the leaf structure but also contributes to desirable aroma and flavor during combustion (Zhu et al., 2014). Nitrogenous compounds, such as proteins and amino acids have complex roles in tobacco quality (Chaplin, 1975). These compounds assess the strength, smoking, and blending qualities. In general, nitrogenous chemicals were thought to have an inverse relationship with quality (Mendell et al., 1984). A lower nitrogen level typically indicates a lighter and less desirable taste. Phenols also influence smoke flavor, quality, and scent thus acting as flavoring precursors. For instance, chlorogenic acid and rutin positively correlate with the quality of flue-cured tobacco. Tobacco ash contains minerals such as calcium, potassium, magnesium, chlorine, phosphorus, and sulphur affect the burning properties (Leffingwell, 1999). For instance, magnesium and potassium accelerate the burn rate, while phosphorus and chloride slow it down (Camlica and Yaldiz, 2021). Additionally, pigments such as lutein, β-carotene, and xanthophyll, serve as the precursors for volatile aroma compounds. These pigments degrade during curing to enhance tobacco flavor. Carboxylic acids including citric, malic, oxalic, and malonic acids also play a role with an inverse relationship observed between citric and oxalic levels and smoking quality (Leffingwell, 1999). This complex interrelationship between alkaloids, carbohydrates, structural components, nitrogenous compounds, minerals, and organic acids highlights the complexity of tobacco leaf composition, making it the primary objective of tobacco breeders.

Due to the complex genetic architecture and the quantitative nature of leaf chemistry traits, most QTL studies in tobacco have focused on simpler traits, such as disease resistance (Cheng et al., 2019), and agronomic traits including yield (Cheng et al., 2015; Ikram et al., 2022a; Liu et al., 2022; Tong et al., 2024). Consequently, there is limited QTL information available on leaf chemistry traits (Julio et al., 2006; Tong et al., 2020, 2025). Therefore, additional research is required to develop more molecular markers, discover more QTLs, and identify genes carrying valuable alleles to elucidate the genetic architecture of these traits.

In the present study, QTL mapping was conducted using multi-environment phenotypic data and a genetic linkage map integrated with SNP-InDel-SSR (Tong et al., 2023). This revealed 18 QTLs with additive individual effects and 3 QTLs with epistatic interactions. QTL mapping combined with bioinformatics and association analysis pinpointed 3 candidate genes showing significant association with total plant alkaloid (TPA), total sugar (TS), reducing sugar (RS) and starch (STA). These findings provide new insights into the genetic basis of tobacco leaf composition traits and offer valuable resources for genomics-assisted breeding to improve the quality of smoking products.

## 2 Materials and methods

### 2.1 Plant material and experimental design

The recombinant inbred line (RIL_F_7_) population was derived from two parental lines, Y3 and K326 through the single-seed descent method. This population contains 271 genotypes and was planted at Shilin in a completely randomized design in 2020, 2021, and 2022 years, which were treated as three distinct environments. The evaluated traits for each genotype included total nitrogen % (TN), potassium % (POT), chlorine % (CHL), reducing sugar % (RS), total plant alkali % (TPA), total sugar % (TS), starch % (STA), chlorogenic acid mg/g (CHA), rutin mg/g (RU), fructose % (FRUC), xanthophyll μg/g (XAN), beta-carotene μg/g (BCA), citric acid mg/g (CA), petroleum ether % (PE), cellulose % (CE), the difference between two sugars % (DS), total amino acids (TAA), aspartic acid (APA), phenylalanine (PA), glutamine (GL), and protein % (PRO). These traits were quantified using high-performance liquid chromatography (HPLC) following the procedures detailed in (Julio et al., 2006; Pang et al., 2006; Jing et al., 2016; Wang P. et al., 2024). Eighteen chemical traits were evaluated in E1 (2020-SL), nine traits in E2 (2021-SL), and eleven traits in E3 (2022-SL).

### 2.2 Statistical analysis

We estimated variance components using the following mixed linear model (Tong et al., 2025).


$$Y_{kh}=\mu +g_{k}+e_{h}+\epsilon_{kh}$$

In this model, $Y_{kh}$ represents the phenotypic value of the *k*-th genotype in the *h*-th environment; μ represents the population mean; $g_{k}$ indicates the genotypic value of the *k*-th genotype, $g_{k}\sim N(0,\;\sigma_{g}^{2})$ ; $e_{h}\sim N(0,\sigma_{e}^{2})$ , denotes the effect of the *h*-th environment; $\epsilon_{kh}\sim N(0,\sigma_{\epsilon }^{2})$ , denotes the residual effect of the *k*-th genotype in the *h*-th environment. To estimate variance components ( ${\hat{\sigma }}_{g}^{2}$ , ${\hat{\sigma }}_{e}^{2}$ , ${\hat{\sigma }}_{\epsilon }^{2}$ ), *mmer* module of the *Sommer* R package was utilized and genotypic values were predicted using the best linear unbiased prediction (BLUP) method. Broad sense heritability was calculated using the formula $H^{2}={\hat{\sigma }}_{g}^{2}/({\hat{\sigma }}_{g}^{2}+{\hat{\sigma }}_{\epsilon }^{2})$ , here ${\hat{\sigma }}_{g}^{2}$ represents the genotypic variance and ${\hat{\sigma }}_{\epsilon }^{2}$ represents residual variances (Tong et al., 2025). Additionally, the pearson correlation coefficient between traits was computed using the *rcorr* function of the *Hmisc* R package. Phenotypic correlation coefficients were calculated separately for each environment, while the genetic correlation coefficients were derived from predicted genotypic values using the BLUP method. Finally, the relationship between traits was visualized using the *corrplot* package in R.

### 2.3 Genotyping and genetic linkage map

A total of 274 samples, including two parental lines, one F_1_ hybrid, and 271 F_7_ individuals, were genotyped using the BIGSEQ-500 platform, following the protocol outlined in our previous study (Tong et al., 2023). High-quality reads were filtered and aligned to the reference genome Nitab4.5 (Edwards et al., 2017) using bioinformatics tools. SNPs and InDels were called out using GATK, with stringent quality control filters. From these data, 46,324 bin markers were constructed and used to develop a high-density linkage map. This map spans a total genetic distance of 3334.88 cM across 24 linkage groups (LGs), with an average marker interval of 0.469 cM (**Supplementary Table S1**).

### 2.4 Genetic and statistical model for QTL mapping

We employed a full QTL model to investigate the genetic architecture of complex traits across multi-environment field experiments. This model incorporates the individual additive genetic effect (*a*) of each QTL, the additive-by-additive epistatic effect of each QTL pair (*aa*), and their interaction with the environments (*ae* and *aae*). We assume ‘*s*’ is the number of segregating QTLs and ‘*t*’ denotes the number of QTL pairs exhibiting epistasis. Then, the phenotypic value of the *k*-th genotype in the *h*-th environment can be described by the following mixed linear model (Tong et al., 2023):


$$y_{kh}=\mu +\sum_{i=1}^{s}a_{i}x_{ik}+\sum_{\begin{array}{c} i,j\in \{1,2.,s\}\\ i\neq j \end{array}}^{t}aa_{ij}x_{ik}x_{jk}+e_{h}+\sum_{i=1}^{s}ae_{hi}u_{hik}+\sum_{\begin{array}{c} i,j\in \{1,2.,s\}\\ i\neq j \end{array}}^{t}aae_{hij}u_{hijk}+\epsilon_{kh}$$

Where, μ is the population mean; *a_i_* is the additive effect of the *i-*th QTL with coefficient $x_{ik}$ which is treated as a fixed effect and takes values 1 and −1 for QQ and qq genotypes of QTL, respectively. Similarly, $aa_{ij}$ is the additive-by-additive epistatic effect of the *i*-th and the *j*-th QTL with coefficient $x_{ik}x_{jk}$ as a fixed effect; $e_{h}$ is the random effect of the *h*-th environment, $e_{h}\sim \;(0,\sigma_{e}^{2})$ ; $ae_{hi}$ indicates the additive by environment interaction effect of the *i*-th QTL and the *h*-th environment with coefficient $u_{hik}(=x_{ik})$ , $ae_{hi}\sim (0,\sigma_{ae_{i}}^{2})$ ; $aae_{hij}$ refer to the interaction effect of the *h*-th environment with $aa_{ij}$ , with coefficient $u_{hijk}(=x_{ik}x_{jk})$ , $aae_{hij}\sim (0,\;\sigma_{aae_{ij}}^{2})$ ; $\epsilon_{kh}$ is the random residual effect, $\epsilon_{kh}\sim \;(0,\sigma_{\epsilon }^{2})$ .

**Figure 1:**
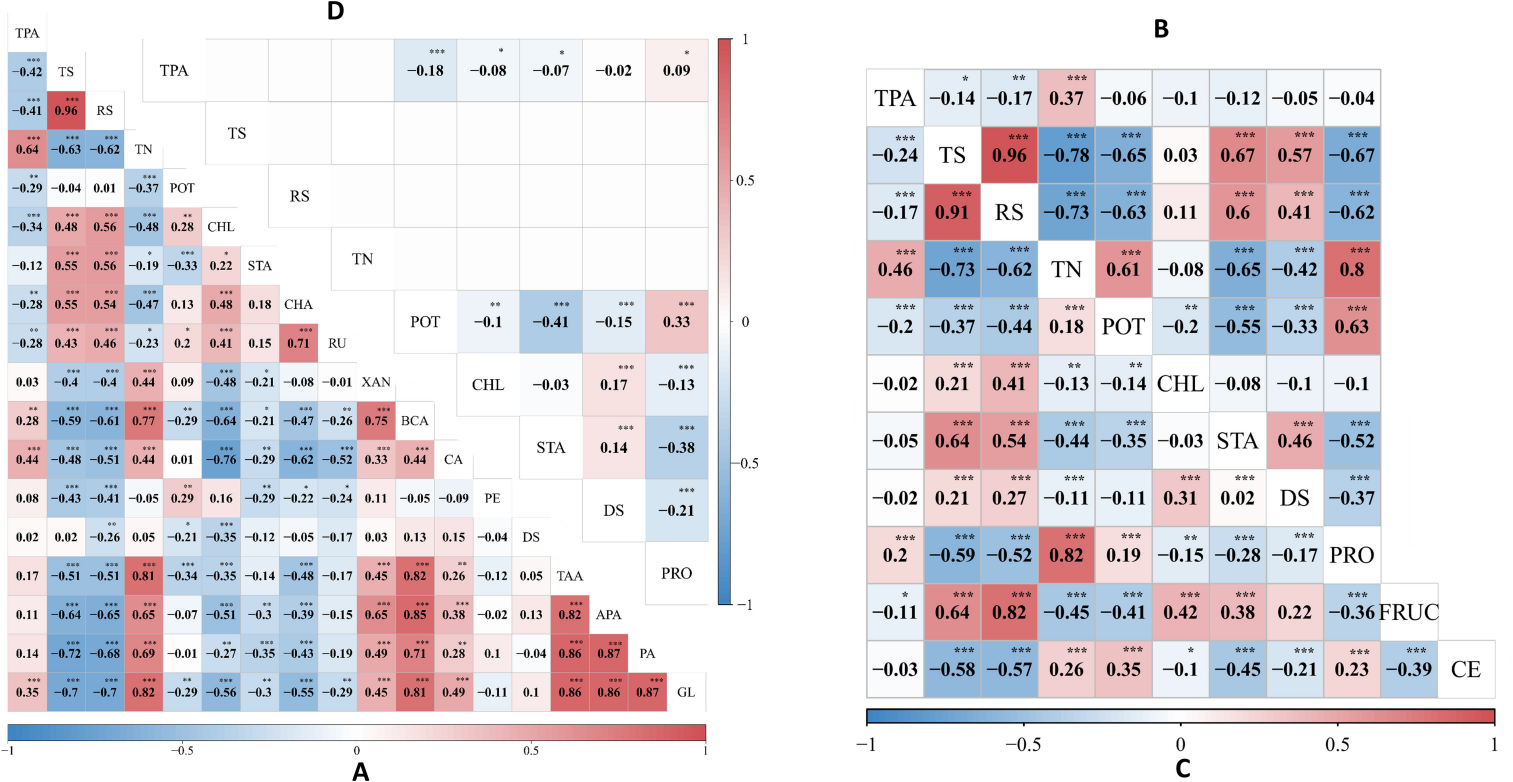
Phenotypic and genotypic correlation analysis across three environments. **(A)** displays the phenotypic correlation coefficient between 18 traits in E1 (2020-SL), **(B)** illustrates the phenotypic correlation coefficient between 9 traits in E2 (2021-SL) and **(C)** shows the phenotypic correlation coefficient between 11 traits in E3 (2022-SL), while **(D)** exhibits a genotypic correlation. Asterisks (^*^, ^**^, and ^***^), denote the significance level at 0.05, 0.01, and 0.001, respectively.

**Figure 2:**
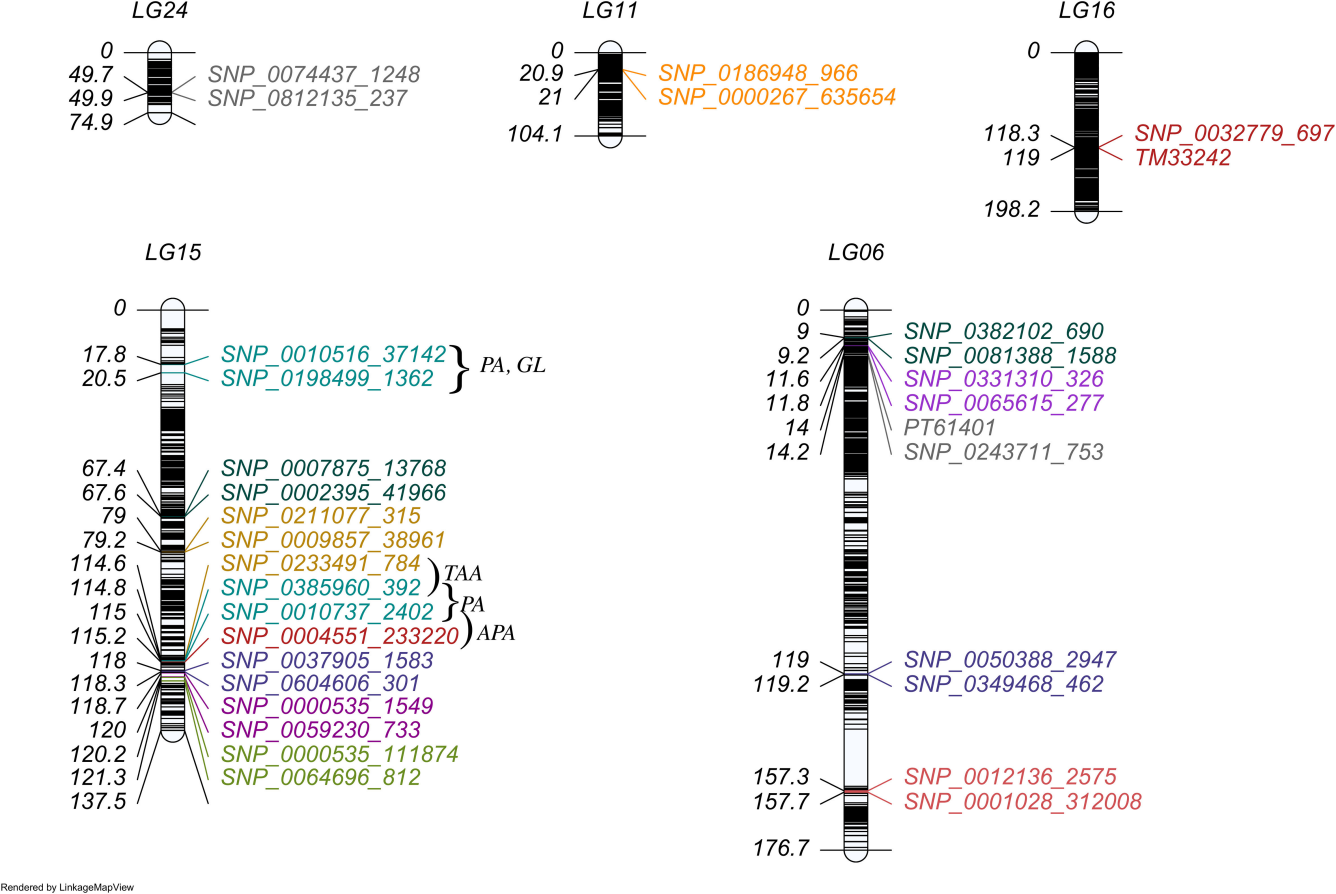
The distribution of QTLs with individual effect in linkage map. Each marker interval represents each QTL region for a specific trait indicated by a corresponding color. The traits were colored as CHA (orange), RU (red salmon), XAN (slate blue), BCA (olive drab), CA (teal), PE (grey), CE (orchid), TAA (golden rod), APA (firebrick), PRO (magenta), While the pleiotropic QTLs (affecting PA, GL) were marked in dark cyan.

QTL analysis was conducted using QTLNetwork 2.0 (Yang et al., 2008), specifically designed for mixed-linear-model-based composite interval mapping (MCIM). Both one-dimensional (1D) and two-dimensional (2D) genome-wide scans were conducted at a walking speed of 1 cM, To control experiment wise type 1 error rate, a critical F-value based on Henderson method III which was determined by permutation testing 1000 times for each tested locus at a significance level of 0.05. The full QTL model was employed to estimate and test the QTL effects and their significance using the Markov Chain Monte Carlo (MCMC) method. Finally, the distribution of QTLs across linkage groups (**Figure 1**, **Figure 2**) was visualized using the *LinkageMapView* package in R.

### 2.5 Prediction of candidate genes

Each QTL region was defined by the two flanking bin markers in the genetic linkage map. The sequences of these markers were aligned to the Nitab4.5 reference genome using Burrow Wheeler Aligner with the *mem* algorithm (Li and Durbin, 2009) and the genes were extracted using *intersect* function in BEDTools (Quinlan and Hall, 2010). Variants including SNPs and Indels were annotated with SnpEff (Cingolani et al., 2012) and those predicted to have moderate to high impact on protein function were retained for further analysis. These variants were subsequently validated through single marker association analysis using PLINK (Purcell et al., 2007), applying a significance threshold of *p* < 0.05. For functional enrichment analysis, the protein sequence of Nitab4.5 reference genome was uploaded to eggnog-mapper website. Finally, Gene Ontology (GO) and KEGG pathway enrichment analysis were performed using *clusterprofiler* R package (Yu et al., 2012). Putative candidate genes were functionally characterized using the *BLASTp* module of NCBI against the non-redundant (nr) protein database.

## 3 Results

### 3.1 Phenotypic evaluation, heritability, and trait correlation analysis

For multi-environment phenotypes, variance components and broad-sense heritability were estimated (**Table 1**). The heritability of traits TS, RS, TN, and POT was 0, indicating that these traits were predominantly influenced by environmental and error variance. In contrast, traits DS and TPA were significantly influenced by genetic factors, as reflected by their heritability of 75.71% and 44.83%, respectively. The majority of traits exhibited statistically significant but negative correlation values (α=0.05) (**Figure 1**). Significant phenotypic correlation was observed between TS and RS across all environments, followed by APA and PA in E1 (**Figure 1A**). The correlation between PA and GL in E1 exhibited a similar trend, as did TN and PRO in E2 and E3, respectively (**Figures 1B, C**). In contrast, a consistently large negative correlation was observed between TS and TN across all environments, though the magnitude varied. The phenotypic correlation between CHL and STA was positive and significant (α=0.05) in E1 but negative and non-significant in E2 and E3. Overall, the correlation pattern varied slightly across environments but remained consistent with general trends. The highest genetic correlation coefficient of 0.33 was observed between POT and PRO (**Figure 1D**), accompanied by a substantial phenotypic correlation in E2 and E3 (**Figures 1B, C**). Additionally, the traits TS, RS, and TN exhibited no genetic correlation, demonstrating that environmental influences drove phenotypic variation rather than shared genetic architecture.

**Table 1:** Summary of variance analysis of 9 chemical traits across 3 environments.

Traits ^a^	Variance components $\left(\sigma^{2}\right)$ ^b^			Heritability ^c^ (%) $H_{g}^{2}$
	$\sigma_{g}^{2}$	$\sigma_{e}^{2}$	$\sigma_{\in }^{2}$	
TN	0	0.03	0.07	0
POT	0.03	0.07	0.08	30
CHL	0.05	0.28	0.22	15.15
RS	0	8.05	24.7	0
TPA	0.13	0.16	0.43	44.83
TS	0	22.89	39.04	0
STA	0.44	0.65	2.5	40.37
DS	13.06	4.19	2.73	75.71
PRO	0	7.95	0.33	0

^1 a^Traits abbreviations, TPA, Total Plant Alkali; TS, Total Sugar; RS, Reducing Sugar; TN, Total Nitrogen; POT, Potassium; CHL, Chlorine; STA, Starch; DS, Difference Between two Sugars; PRO, Protein.

^b^Variance Components $\left(\sigma^{2}\right)$ : $\sigma_{g}^{2}$ for genotypic variance, $\sigma_{e}^{2}$ for environmental variance, and $\sigma_{\text{ϵ}}^{2}$ for residual variance.

^c^Heritability: $H_{g}^{2}(\%)$ is the general heritability calculated by $H_{g}^{2}=\sigma_{g}^{2}/(\sigma_{g}^{2}\;+\;\sigma_{e}^{2})$ .

### 3.2 QTL distribution on linkage groups

**Figure 3:**
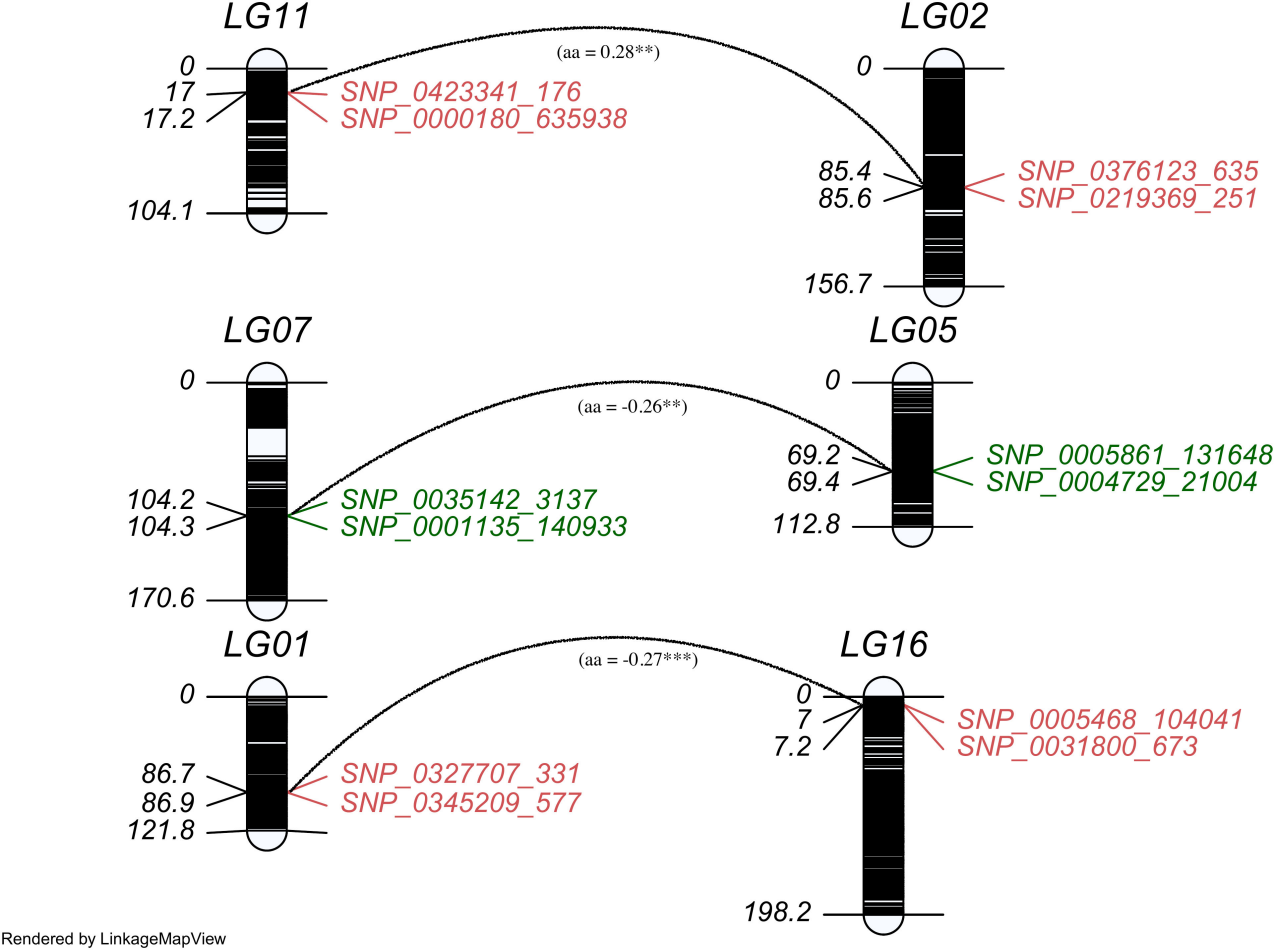
The distribution of QTLs contributing epistatic effects in the linkage map. Each paired epistatic QTL was indicated by two marker intervals connected with an arc line with its magnitude and marked the same color for one trait, red for chlorogenic acid (CHA) and green for rutin (RU).

We identified 18 QTLs associated with 12 traits that exhibited significant individual additive effects across 5 linkage groups (**Figure 2**) and three pairs of epistatic QTLs distributed over 6 linkage groups for two traits (**Figure 3**). Two epistatic QTL pairs were detected for chlorogenic acid (CHA) and one QTL pair for rutin (RU). LG15 contained the highest number of QTLs with individual additive effects (10 QTLs), followed by LG06 with 5 QTLs. LG11, LG16, and LG24 each carried one QTL.

### 3.3 Additive interaction effect, heritability, and pleiotropic effects

A total of 18 QTLs with additive (*a*) effects were detected for 12 traits (**Table 2**). Out of these 18 QTLs, 11 QTLs contributed positive additive effects, while 7 QTLs exhibited negative additive effects, indicating the complex genetic architecture (**Supplementary Figure S1**). The heritability of each QTL explained a percentage of the phenotypic variation, ranging from 2.21% to 20.05%. The majority of the QTLs had small additive effects and lower heritability, thus regarded as minor-effect QTLs. The QTLs *qAPA16-247* $\left(h_{a}^{2}=20.05\%\right)$ , *qGL15-18* $\left(h_{a}^{2}=18.85\%\right)$ , *qAPA15-250* $\left(h_{a}^{2}=15.75\%\right)$ , *qPA15-249* $\left(h_{a}^{2}=12.2\%\right)$ , and *qTAA15-248* $\left(h_{a}^{2}=11.16\%\right)$ were identified as major-effect QTLs due to their significant and larger contribution to phenotypic variation. However, *qTAA15-248*, *qTAA15-169*, *qGL15-18, qAPA15-250*, *qPA15-249*, and *qPA15-18* exhibited substantial additive effects on their respective traits and were located on LG15. For breeding purposes, one of the main goal is to identify QTLs that express stably across environments with minimal or non-significant QTL-environment interactions. In our study, all QTLs exhibited no interaction effects, indicating they can be utilized in breeding new variety for most general environment. Notably, *qPA15-18* and *qGL15-18* were located in a same marker interval ranged by SNP_0010516_37142 and SNP_0198499_1362. The co-localizations indicate there may be gene which take pleiotropic effect on PA and GL. This hypothesis was further supported by the substantial and reasonably high estimated correlation coefficient of 0.87 between PA and GL (**Figure 1A**).

**Table 2:** Additive effect and heritability of QTL for 12 traits.

Trait	*QTL*	M_-_	M_+_	Position	Support interval	*a*	$h_{a}^{2}(\%)$
				cM	cM		
CHA	*qCHA11-69*	SNP_0186948_966	SNP_0000267_635654	21	20-22	-0.34^***^	2.21
RU	*qRU6-377*	SNP_0012136_2575	SNP_0001028_312008	157.7	157-158	0.39^***^	4.91
XAN	*qXAN6-336*	SNP_0050388_2947	SNP_0349468_462	119.2	117-121	-2.86^***^	3.62
	*qXAN15-258*	SNP_0037905_1583	SNP_0604606_301	118.3	116-119	3.71^***^	6.11
BCA	*qBCA15-263*	SNP_0000535_111874	SNP_0064696_812	121.3	118-122	1.77^***^	6
CA	*qCA6-25*	SNP_0382102_690	SNP_0081388_1588	9.2	9-9.2	0.086^***^	8.91
	*qCA15-140*	SNP_0007875_13768	SNP_0002395_41966	67.6	67-70	-0.08^***^	6.93
PE	*qPE6-47*	PT61401	SNP_0243711_753	14.2	13-14.2	-0.16^***^	5.66
	*qPE24-87*	SNP_0074437_1248	SNP_0812135_237	49.9	48-55	-0.10^***^	2.24
CE	*qCE6-36*	SNP_0331310_326	SNP_0065615_277	11.8	11-12	-0.44^***^	11.8
TAA	*qTAA15-169*	SNP_0211077_315	SNP_0009857_38961	79.2	74-84	1127.40^**^	6.85
	*qTAA15-248*	SNP_0233491_784	SNP_0385960_392	114.8	113-117	1438.88^***^	11.16
APA	*qAPA15-250*	SNP_0010737_2402	SNP_0004551_233220	115.2	113-117	35.56^***^	15.75
	*qAPA16-247*	SNP_0032779_697	TM33242	119	118-119	-40.12^***^	20.05
PA	*qPA15-18*	SNP_0010516_37142	SNP_0198499_1362	20.5	17-25	25.80^***^	9.76
	*qPA15-249*	SNP_0385960_392	SNP_0010737_2402	115	113-117	28.85^***^	12.2
GL	*qGL15-18*	SNP_0010516_37142	SNP_0198499_1362	20.5	17-25	211.43^***^	18.85
PRO	*qPRO15-261*	SNP_0000535_1549	SNP_0059230_733	120	118-121	0.14^***^	4.98

^2^ a are the additive effect; M_-_ and M_+_ are the left and the right flanking markers of the QTL, markers with the prefix PT and TM are SSRs; $h_{a}^{2}(\%)$ is the heritability due to the additive effects. *, ** and *** represents the significance level at 0.05, 0.01 and 0.001, respectively.

### 3.4 Additive-additive epistatic effect and heritability

A two-dimensional (2D) genome wide scan detected three epistatic QTL pairs associated with chlorogenic acid and rutin across LG1/LG16, LG2/LG11, and LG5/LG7 (**Table 3**). All these QTL pairs exhibited minimal additive-additive epistatic effects. Furthermore, these epistatic QTLs exhibited no significant additive-additive epistasis by environment interaction effects. Each QTL pair explained less than 3% of the overall phenotypic variation. Notably, the QTL pair *qRU5-182*/*qRU7-131* accounted for greater heritability $\left(h_{aa}^{2}=2.37\%\right)$ than *qCHA1-216*/*qCHA16-21* $\left(h_{aa}^{2}=1.45\%\right)$ and *qCHA2-281*/*qCHA11-56* $\left(h_{aa}^{2}=1.29\%\right)$ . Interestingly, the interaction effects of *qCHA2-281*/*qCHA11-56* and *qRU5-182*/*qRU7-131* were -0.26 and -0.27, respectively (**Supplementary Figure S2**). This indicated that the genotype of two QTL from same parent will reduce the trait value, in contrast, the genotype from different parent will increase the trait values.

**Table 3:** The additive-additive epistatic effect and heritability of QTL for chlorogenic acid and rutin.

Trait	QTL*_i_*	M_-_	M_+_	Position*_i_*	Support interval	QTL*_j_*	M_-_	M_+_	Position_j_	Support interval	*aa*	$h_{aa}^{2}\;(\%)$
				cM	cM				cM	cM		
CHA	*qCHA1-216*	SNP_0327707_331	SNP_0345209_577	86.9	86.7-87.3	*qCHA16-21*	SNP_0005468_104041	SNP_0031800_673	7.2	4.8-7.6	0.28^**^	1.45
	*qCHA2-281*	SNP_0376123_635	SNP_0219369_251	85.9	85.4-85.8	*qCHA11-56*	SNP_0423341_176	SNP_0000180_635938	17.2	16.2-18.1	-0.26^**^	1.29
RU	*qRU5-182*	SNP_0005861_131648	SNP_0004729_21004	69.4	68.1-71.6	*qRU7-131*	SNP_0035142_3137	SNP_0001135_140933	104.3	100.8-104.5	-0.27^***^	2.37

aa is the additive-additive epistaitic effect; M_-_ and M_+_ are the left and the right flanking markers of the QTL, $h_{aa}^{2}(\%)$ is the heritability due to the aa effects. *, ** and *** represents the significance level at 0.05, 0.01 and 0.001, respectively.

### 3.5 Candidate gene prediction through association and enrichment analysis

**Figure 4:**
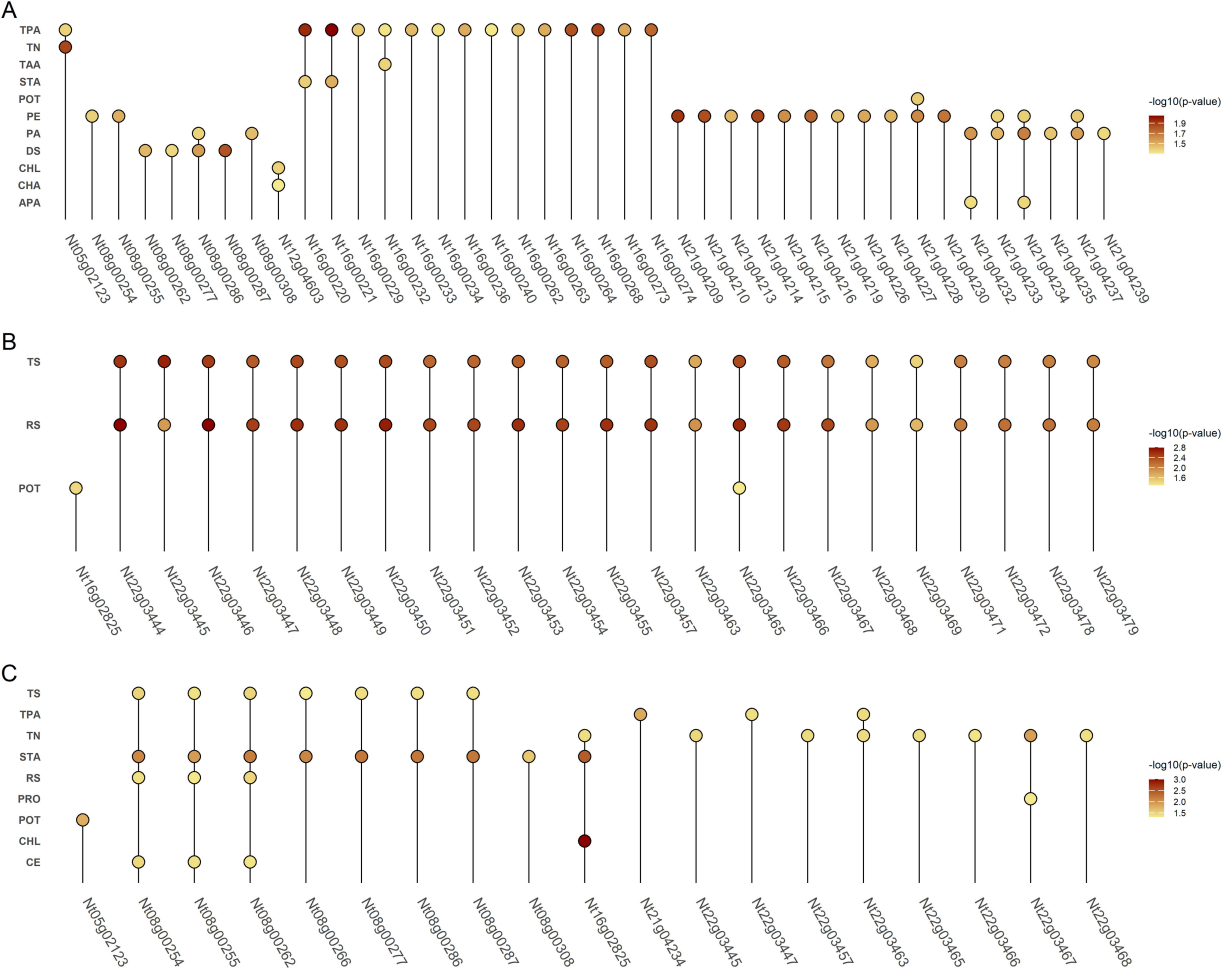
Dot plots showing genes exhibiting significant associations with multiple phenotypes across three environments. Panel A represents Environment 1, Panel B Environment 2, and Panel C Environment 3. The color intensity of each dot indicates the -log_10_ of the p-value for the association.

**Figure 5:**
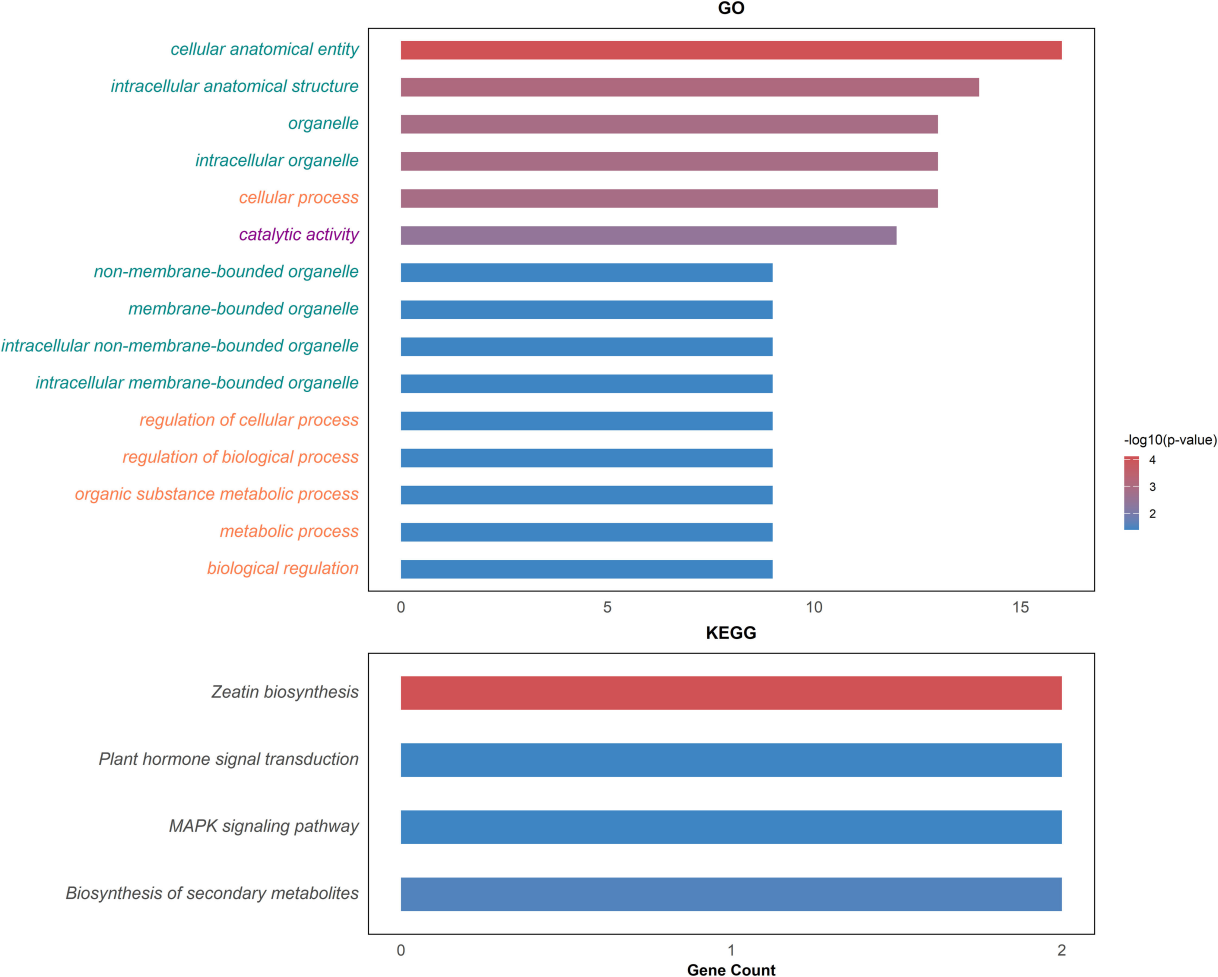
Top significantly enriched GO terms and KEGG pathways. The green colored of GO terms represents cellular process, orange colored denotes biological process and magenta colored is for molecular function.

Through comparative mapping with the Nitab4.5 reference genome, the QTLs were mapped onto ten chromosomes, namely Nt05, Nt06, Nt08, Nt10, Nt12, Nt16, Nt18, Nt21, Nt22 and Nt24. A total of 477 genes were identified from these QTL regions (**Supplementary Table S2**). 56,983 variants within these genic regions were identified and annotated through the SnpEff tool. Wherein, 395 variants in 99 genes were determined to have moderate to high impacts on the protein level and were filtered for further analysis (**Supplementary Table S3**). These 99 genes were subjected to genome-wide association study using single marker association analysis. This analysis revealed 205 variants in 66 genes demonstrating significant association with multiple phenotypes across all the environments (**Figure 4**), (**Supplementary Table S4**). Among the 66 genes, 20 were significantly enriched in six GO biological processes, eight in GO cellular components, and one in GO molecular functions, while three genes were enriched in four KEGG pathways (**Figure 5**). Based on these results, *Nt08g00266, Nt16g00236*, and *Nt22g03479* were predicted as candidate genes. GWAS analysis further revealed that *Nt08g00266* was significantly associated with total sugar TS and STA, *Nt16g00236* with TPA, and *Nt22g03479* with TS and RS. The functions of these candidate genes were retrieved from BLASTp. This analysis revealed that *Nt16g00236* encodes a mitogen-activated protein kinase (MAPK), *Nt22g03479* encode scopoletin glucosyltransferase, and *Nt08g00266* encodes a MYC2-like transcription factor.

## 4 Discussion

Tobacco leaf chemical traits are quantitative in nature and governed by the combined effects of multiple genes. Their complex genetic architecture and susceptibility to environmental influences made traditional breeding methods based on phenotypic selection ineffective for improving these traits (Tong et al., 2020). Thus, understanding the genetic architecture of these complex traits is crucial for designing efficient breeding programs to improve tobacco leaf chemical traits.

Molecular markers played a key role in exploring the genetic basis of complex quantitative traits (Julio et al., 2006). Compared to other solanaceous crops like potato (Tanksley et al., 1992), tomato (Haanstra et al., 1999; Tanksley et al., 1992), and pepper (Lefebvre et al., 1995), tobacco has fewer molecular resources available for genetic mapping (Tong et al., 2021). Genetic maps in these crops have greatly facilitated QTL analysis. In tobacco, various types of molecular markers, including AFLPs (Julio et al., 2006; Moon and Nicholson, 2007), SSRs (Tong et al., 2016, 2024), InDels and SNPs (Song et al., 2015; Xiao et al., 2015; Tong et al., 2023, 2025; Xu et al., 2024) have been employed to construct a genetic linkage map. Among these, SNPs are the most widely used markers due totheir dense distribution across the genome (Tong et al., 2025). However, there are limited QTLs identified for tobacco leaf composition traits. This is in part due to its larger genome size (4.3 Gb) along with narrow genetic diversity (Ikram et al., 2022; Wang J. et al., 2024).

The first QTL study on traits related to tobacco leaves and smoke traits was reported in 2006 (Julio et al., 2006). In that study, a partial genetic linkage map was constructed utilizing 138 low-throughput markers, including AFLP, ISSR, SSAP, and SCAR, covering 18 linkage groups. Their study identified six QTLs associated with total alkaloids, proline, and reducing sugars in an RIL population. In contrast, our study identified distinct QTLs for proline and discovered new QTLs associated with CHA, RU, XAN, BCA, CA, PE, TAA, APA, PA, GL, and CE. These findings provided new QTL resources for the genetic improvement of tobacco chemical traits. Compared with previous QTL studies on leaf chemistry, our research has several advantages. First, we used a high-density linkage map with 46,324 markers across 24 linkage groups, representing the most saturated linkage map to date. Second, we employed a full QTL model which included not only additive effects but also additive-additive epistatic interaction and their interaction with the environment as well. Consequently, we identified 18 QTLs with significant individual effects underlying 12 traits and three pairs of epistatic QTLs. Notably, no QTL-environment interactions were observed for any of the identified QTLs. A significant finding of our study was that *qPA15-18* and *qGL15-18* were located in the same linkage group and likely indicated the existence of pleiotropic QTLs. On the other hand, no QTLs were identified for nine traits (**Table 1**); which might be due to the absence of genotypic variation or the traits are controlled by many minor-effect genes that couldn’t be detected by traditional mapping method (Heffner et al., 2009; Xu et al., 2024).

In this study, GO enrichment analysis indicated that the genes were significantly enriched in terms related to metabolic process, organic substance metabolic process, catalytic activity, regulation of cellular process, and biological regulation. These findings suggest that key genes are involved in the biosynthesis, metabolism, and modification of essential compounds such as alkaloids, sugars, and phenolics, which are the primary determinants of tobacco leaf composition. Metabolic processes are crucial regulators of nicotine biosynthesis (Qin et al., 2020; Shoji et al., 2024). The enrichment of catalytic activity highlights a functional emphasis on enzymatic processes, including the breakdown of starch (Ye et al., 2024), synthesis of secondary metabolites (Malinowski et al., 2007), and degradation of nicotine (Li et al., 2024). Overall, these enriched biological functions collectively contribute to shaping the chemical composition of tobacco leaves.

By integrating the results from linkage mapping, association analysis, and enrichment analysis, three candidate genes *Nt08g00266*, *Nt16g00236*, and *Nt22g03479* were identified. Functional annotation using the *BLASTp* tool revealed that *Nt08g00266* encodes a MYC2-like transcription factor, previously reported to regulate carbohydrate metabolism and pollen development via the jasmonic acid (JA) signaling pathway in tobacco (Bian et al., 2022). Consistent with this, our KEGG enrichment analysis indicated that *Nt08g00266* is involved in the Plant hormone signal transduction pathway. *Nt16g00236* encodes a mitogen-activated protein kinase (MAPK), showing homology to serine/threonine kinases in *Nicotiana tabacum* (Wilson et al., 1995). MAPKs are known to participate in signal transduction, autophosphorylation, substrate phosphorylation, and disease resistance mechanisms. Furthermore, *Nt22g03479* encodes a scopoletin glucosyltransferase which promotes the glucosylation of scopoletin, a process critical for the accumulation of scopoletin and scopoline. These secondary metabolites enhance the antiviral defense mechanisms in response to tobacco mosaic virus, by reducing reactive oxygen intermediates and improve plant resilience (Chong et al., 2002). Based on its role in secondary metabolite biosynthesis, we formulated that *Nt22g03479* plays a regulatory role in plant defense mechanisms by modulation of secondary metabolites (Siwinska et al., 2014).

In our study, candidate genes were predicted based on the sequences of chromosome region of QTL. Although, we performed association analysis and enrichment analysis to strengthen the reliability of these candidate genes but still functional validation of these candidate genes was required for their application in advanced molecular and biological techniques.

## 5 Conclusion

In conclusion, 21 leaf composition traits were studied through QTL mapping. Our analysis revealed 18 QTLs exhibiting significant individual additive effects, of which only one QTL (*qPA15-118* & *qGL15-18*) exhibited pleiotropic effects, along with 3 pairs of epistatic QTLs. Prioritization of candidate genes was achieved through subsequent association, GO and KEGG enrichment analysis and as a result, *Nt08g00266*, *Nt16g00236* and *Nt22g03479* were mined as candidate genes. These candidate genes are implicated in critical biological processes, functions in mitogen activate protein kinase signalling pathway, carbohydrate metabolism through hormone signalling and biosynthesis of secondary metabolites. This study brought new insights into the genetic architecture of these chemical traits and paved a way to molecular improvement of tobacco leaf chemistry.

## Data Availability

The original contributions presented in the study are included in the article/**Supplementary Material**. Further inquiries can be directed to the corresponding authors.
