# Supplementary figures and images for: Integration of QTL mapping and GWAS reveals the complicated genetic architecture of chemical composition traits in tobacco leaves
Source: Front Plant Sci. 2025 Jun 25;16:1616591. doi: 10.3389/fpls.2025.1616591 (PMC12238100; doi:10.3389/fpls.2025.1616591)

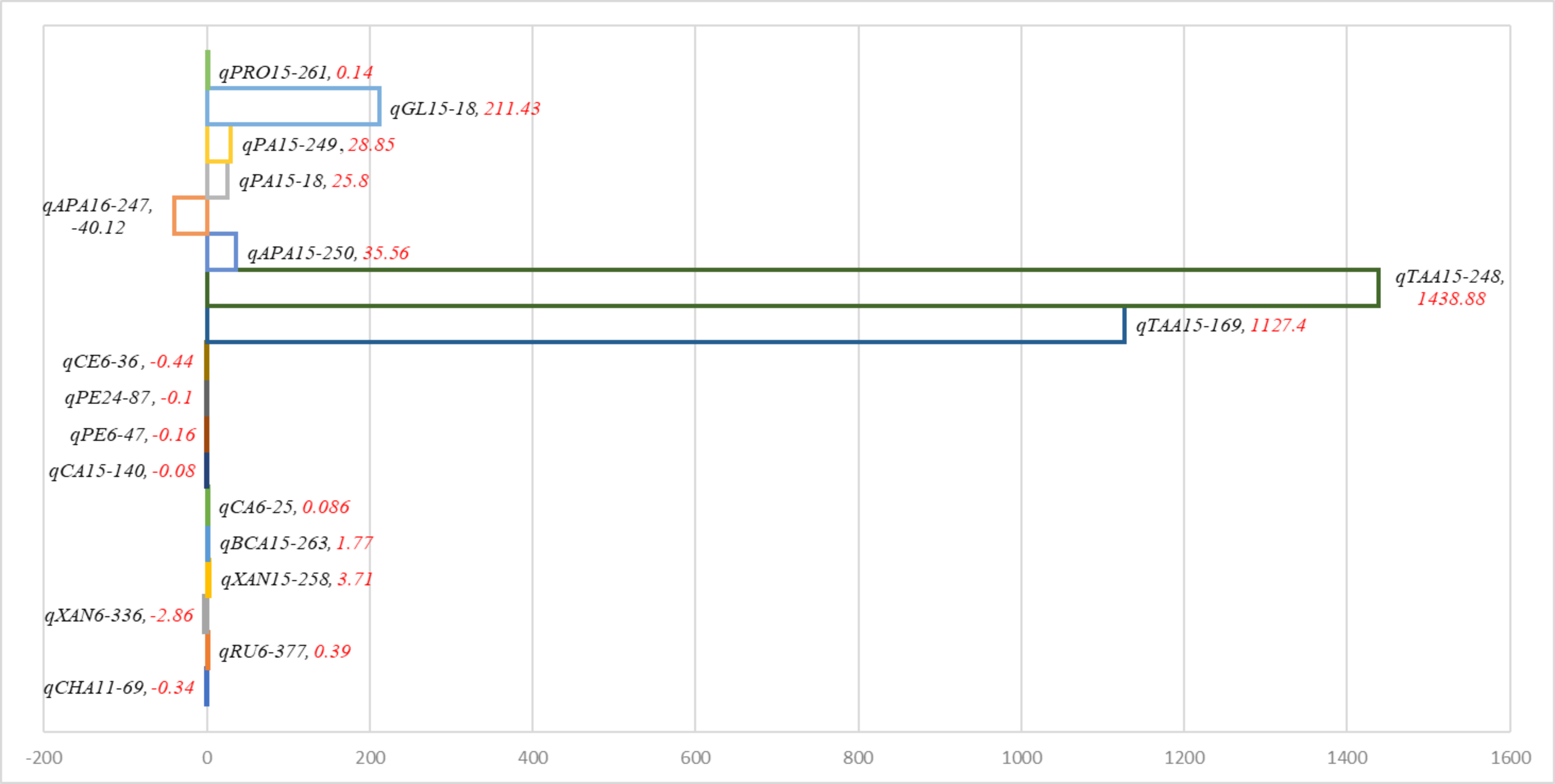

Supplement: Supplementary file 1 [file Image1.tiff]

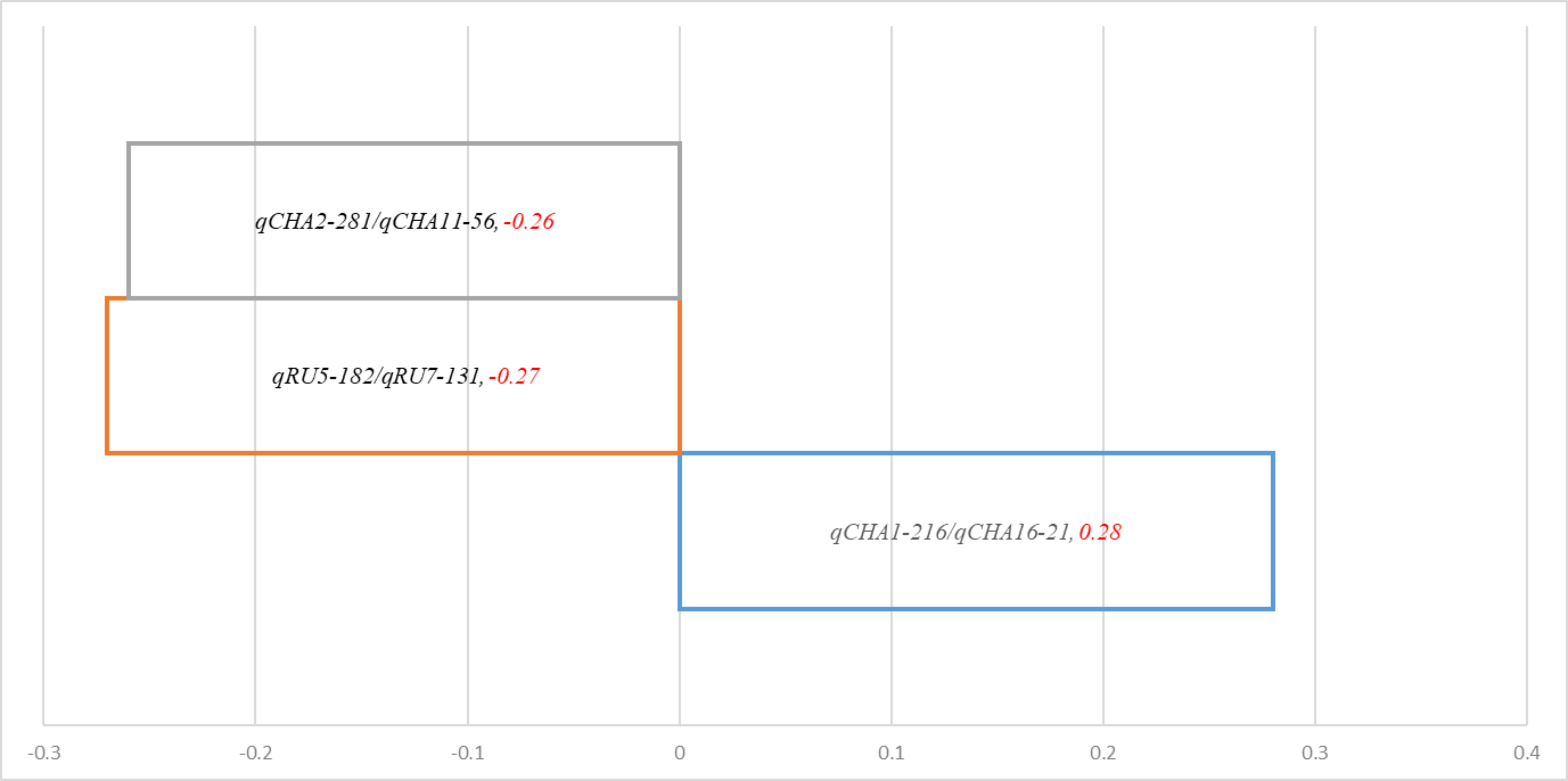

Supplement: Supplementary file 2 [file Image2.tiff]
